# Behavioural Responses of Tropical Bed Bug *Cimex hemipterus* (F.) (Hemiptera: Cimicidae) to Coloured Harbourage

**DOI:** 10.21315/tlsr2024.35.2.13

**Published:** 2024-07-31

**Authors:** Abd Hafis Abd Rahim, Abdul Hafiz Ab Majid

**Affiliations:** 1 Household and Structural Urban Entomology Laboratory, School of Biological Sciences, Universiti Sains Malaysia, 11800 USM Pulau Pinang, Malaysia; 2 Centre for Insect Systematics, Faculty of Science and Technology, Universiti Kebangsaan Malaysia, 43600 UKM Bangi, Selangor, Malaysia

**Keywords:** Tropical Bed Bug, Reproduction, Harbourage Colour, Rearing Behavioural, Oviposition, *Cimex hemipterus*, Pepijat Tropika, Pembiakan, Tempat Berteduh, Tingkah Laku Pembiakan, Oviposisi, *Cimex hemipterus*

## Abstract

Population of the tropical bed bug *Cimex hemipterus* (F.) (Hemiptera: Cimicidae), a temporary ectoparasite on both humans and animals, have surged in many tropical countries. Tropical bed bugs preferences when selecting a suitable harbourage and oviposition site were investigated. Two-choice and three choice colour assays were conducted to determine whether bed bugs will choose black, red or white coloured harbourages. Then, 50 1st instar were reared in containers containing black, red and white (control) paper served as the harbourages and observed for 12 weeks. Both fed and starve male, female and nymph strongly preferred red and black coloured harbourage compared to white coloured harbourage. Oviposition assays showed that female bed bugs prefered to laid their eggs on red coloured harbourages compared to black coloured harbourages. Rearing experiment showed that there was no significant difference (*p* > 0.05) between final population size of tropical bed bug. However, tropical bed bugs reared in container with red paper (600 ± 89.238) have the highest number of individuals followed by black (473 ± 133.841) and white (485 ± 84.234) paper. Bed bug preference towards coloured harbourage provide useful information for those developing new bed bug control method or improving bed bug infestation monitoring devices.

## Abstrak

Populasi pepijat tropika *Cimex hemipterus* (F.) (Hemiptera: Cimicidae), ektoparasit sementara pada manusia dan haiwan, telah meningkat di banyak negara tropika. Keutamaan pepijat apabila memilih tempat berteduh dan oviposisi yang sesuai dikaji. Ujian warna dua pilihan dan tiga pilihan telah dijalankan untuk menentukan sama ada pepijat akan memilih tempat berteduh berwarna hitam, merah atau putih. Kemudian, 50 instar 1 dibesarkan dalam bekas yang mengandungi kertas hitam, merah dan putih (kawalan) yang berfungsi sebagai tempat berteduh dan diperhatikan selama 12 minggu. Kedua-dua jantan, betina dan nimfa yang diberi makan dan kelaparan sangat menyukai tempat berteduh berwarna merah dan hitam berbanding tempat berteduh berwarna putih. Ujian oviposisi menunjukkan bahawa pepijat betina lebih suka bertelur di tempat berteduh berwarna merah berbanding dengan tempat berteduh berwarna hitam. Eksperimen berteduh menunjukkan bahawa tidak terdapat perbezaan yang signifikan (*p* > 0.05) antara saiz populasi akhir pepijat tropika. Walau bagaimanapun, pepijat tropika yang dibiak di dalam bekas dengan kertas merah (600 ± 89.238) mempunyai bilangan individu tertinggi diikuti oleh kertas hitam (473 ± 133.841) dan putih (485 ± 84.234). Keutamaan pepijat terhadap tempat berteduh berwarna memberikan maklumat berguna bagi mereka yang membangunkan kaedah baru kawalan pepijat atau memperbaik peranti pemantauan infestasi pepijat.

**Highlights**

- Two-choice and three choice colour assays were conducted to determine-whether bed bugs will choose black, red or white coloured harbourages.
- Both fed and starved male, female and nymph strongly preferred red and black coloured harbourage compared to white coloured harbourage.
- Oviposition assays showed that female bed bugs preferred to lay their eggs on red coloured harbourages compared to black coloured harbourages.

## INTRODUCTION

Global resurgence of the bed bugs has sparked interest among scientist and pest control companies in developing effective and efficient control and management strategies (Abd Rahim *et al*. 2014; Ab Majid & Zahran 2015; Zahran *et al*. 2016; Lim & Ab Majid 2020). Knowledge of biology and ecology of the bed bug, *Cimex lectularius* or *Cimex hemipterus* under various environmental conditions is crucial to improve the management techniques. Bed bugs colonies are required to be tested and this is proved to be a problem since bed bug is an obligatory blood feeder which mean they are required to be fed on a human or animal host for nutritional and reproduction purposes (Abd Rahim *et al*. 2016; Miller *et al*. 2013; Zorrilla-Vaca *et al*. 2015).

Visual cues are used by insects for a wide variety of behaviours including, but not limited to, finding mates, recognising hosts, seeking shelter and ovipositing. These visual cues are often based on the perception of colour. The ability to distinguish between different wavelengths of light, as opposed to different light intensities, is termed colour vision (Menzel & Backhaus 1991; Cuthill 2006). Many insects exhibit colour vision and colour preferences. Colour preference, which is a receptor-neural strategy (Menzel & Backhaus 1991), is an example of how the images that an insect sees can produce a biologically significant behavioural response. In addition to abiotic factors, such as temperature and moisture (How & Lee 2014), and biological factors, such as the size and development of the compound eyes, responses to light are important for understanding how insects respond to visual stimuli (Weiss 1943).

Early studies by Aboul-Nasr and Erakey (1969) have documented that the common bed bug is able to distinguish between different wavelengths of light. Short-wavelength colours such as violet and bluish-green were preferred compared with the other colours tested. Red had attractive qualities, while yellow appeared to be the least attractive. More recent studies by Singh *et al*. (2015) have also shown that aggregations of specifically adult male bed bugs and third–fifth-instar bed bugs prefer black and red harbourages compared with other tested colours. Additionally, McNeil *et al*. (2016) reported that harbourage colour preferences change according to gender, nutritional status, aggregation and life stage. Female bed bugs prefer harbourages with shorter wavelengths (lilac and violet) compared to males, whereas males prefer harbourages with longer wavelengths (red and black) compared with females. The preference for orange and violet harbourages is stronger when bed bugs are fed as opposed to when they are starved. Bed bug nymphs preferred different coloured harbourages at each stage of development, which is indicative of their developing eye structures and pigments.

The objectives of this study were:

1. To determine whether tropical bed bugs would show significant gender and nutritional status differences in response to different colours when selecting a suitable harbourage.
2. To determine whether female bed bugs would prefer to oviposit eggs on harbourage of specific colours.
3. To determine the effect of coloured harbourages to bed bug’s population.

## MATERIALS AND METHODS

### Bed Bugs Culture and Rearing

The tropical bed bugs, *C. hemipterus*, were reared in Household and Structural Urban Entomology Laboratory, Universiti Sains Malaysia. The samples were originated from specimens collected at Kuala Lumpur International Airport (KLIA) back in 2014. Bed bugs were reared in plastic containers (8 cm in height, 8 cm in diameter) containing folded white A4 paper strips (Double A, Chachoengsao, Thailand) as their harbourage. The paper strips were placed perpendicular to the bottom of the container to provide a surface for the bed bugs to walk and deposit eggs. Plastic containers were covered with a piece of fine net cloth (13 cm × 13 cm) and a rubber band to hold it in place (Abd Rahim *et al*. 2015). The bed bugs were fed weekly on a human volunteer’s arm. Bed bugs were carefully transferred into sample vials which then covered with a fine net cloth and a rubber band to hold it in place. After net cloth was secured, sample vials were placed on the forearm of the volunteer and left for 20 min so that the bed bugs can feed and sucked the blood (Abd Rahim *et al*. 2016). The feeding of bed bugs on human host followed the protocol approved by the Human Research Ethics Committee USM (HREC) with code USM/JEPeM/19120868. The tropical bed bugs colonies were cultured in an incubator with temperature set at 26 ± 1°C, 65 ± 5% relative humidity (RH) and a photoperiod of 12 h: 12 h (light: dark).

Bed bugs in two nutritional stages were used: fed and starved. Starved bed bugs were those that had not been fed within their normal once a week feeding regimen (blood fed 7 days before experimentation). Fed bed bugs were those that had been blood fed 1–2 days before experimentation. Three stages of bed bug were used in this study; male, female and the fifth instar. Every replicate use a new individual which was randomly selected from the rearing containers.

### Visual Arena

The visual bioassays were conducted in a Lab Tek, extra deep, Petri dish (90 by 20 mm; ThermoFisher Scientific, Pittsburgh, PA). The base of each Petri dish was minimally scourged with 60 grit sandpaper to allow bed bugs to walk more easily within the arena without influencing the edge-following behaviour of bed bugs. To prevent positional biases, within each experiment, a clean Petri dish arena was randomly selected and placed within a large metal tray (30 by 30 cm), to further prevent bed bugs from escaping.

**Figure 1:**
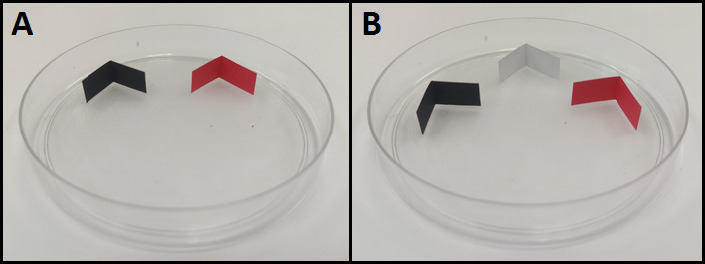
(A) Two colours and (B) three colours choice assays arena.

Each Petri-dish arena contained two or three colour choices (depending on the type of experiment) that were arranged as small L-shaped harbourages and placed perpendicular to the Petri dish floor (Fig. 1). The L-shaped coloured harbourages (2 cm long by 1 cm wide) were made from various coloured A4 paper. All harbourages were under the same light conditions. The experimental room was maintained at 26°C–27°C and average RH of 60.5%. All bed bugs were placed in the experimental room for acclimatisation 24 h prior to the bioassays. As the bed bug harbourage colour choice experiments were conducted, the doors were closed, and no human hosts were present inside the experimental room. Gloves were used in all situations to keep human odours off all harbourages and arenas.

### Colour Choice Assays

Bioassays were performed to determine whether tropical bed bugs would show significant differences in response to various colours when selecting a suitable harbourage. Bed bugs were given the choice of three colour harbourages: red, black and white (control). Two types of experiment were conducted:

1. Two choice harbourage colours (black vs. white, red vs. white and blackvs. red).
2. Three choice harbourage colours (black vs. red vs. white).

Order of the colour harbourages was randomised within each Petri dish. Paper harbourages were placed in the arena 2 cm apart and 1 cm from the perimeter of the Petri dish to prevent edge effects.

A single bed bug was then placed in the middle of the Petri dish arena and was given 10 min to make a choice of climbing to a particular coloured harbourage. This was considered to be one replication. After the end of the 10-min period, the harbourage which the bed bug was found was recorded. After each replicate, new coloured harbourages were placed in the arena and the positions of the control and coloured harbourages were randomised to prevent positional biases. Each coloured harbourage and each bed bug was used only once. This experiment was replicated 40 times with males, females and 5th instar.

### Oviposition Experiment

Experiments were conducted to test if female bed bugs prefer to oviposit eggs on harbourages of specific colours. Three colour harbourages were tested simultaneously (black, red and white). Immediately following the usual feeding regime, female bed bugs were allowed to mate for 4 h. Following mating, 5 female bed bugs were placed in a Petri dish arena with three coloured harbourages listed above and left for 7 days to oviposit. After 7 days, eggs on each coloured harbourage were counted. This oviposition experiments were replicated 10 times (50 females in total) simultaneously.

### Rearing Using Coloured Harborages

**Figure 2:**
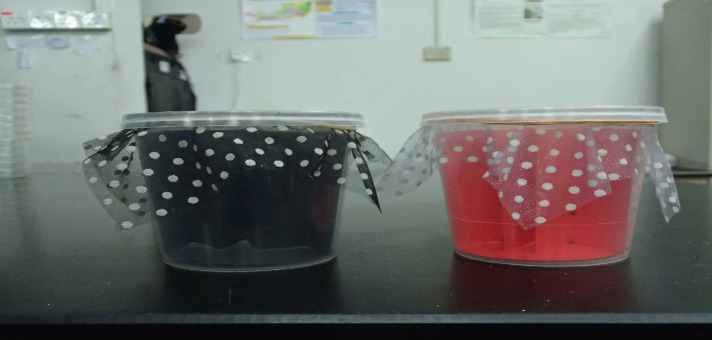
Bed bugs were reared in containers with coloured harbourages.

Ten males and 10 females that had been fed 7 days ago were collected from the colonies and fed through artifical feeding system until they reached engorged weight. All fed adults were placed in a new container and after 7 days, harbourage paper containing eggs were observed until 50 first instars were obtained. The first instars were then transferred to containers with respective coloured harbourage (white as control, red and black), three replicates for each colour (Fig. 2). The physical condition of the container (transparent) and the net covering it (polkadot muslin net) were not changed since the same kind were used for the rearing of the bed bug colonies in Household and Structural Urban Entomology Laboratory since 2014. The only condition that changed was the colour of the harbourages, red and black was added as well as white (as control – white paper has been used as the harbourage since 2014). Colour papers (Benchmark Paper Products Sdn. Bhd., Penang, Malaysia), black, red and white, were cut to 15 cm × 6 cm rectangular shape and circular shape with 5 cm diameter. Circular paper was placed at the bottom of the container and the rectangular paper was folded 8 times so it can fit into the container and placed perpendicular to the circular one at the bottom.

The colonies were fed on a human volunteer for every 7 days. Live individuals were counted before each feeding session. Live individual counted based on their posture and movement in the container. The ones that were in an upside-down position with no signs of movement at all after 10 sec of observation were counted as dead (Ab Majid & Zahran 2017). The number of molted bed bugs were also counted based on how many exoskeleton were presented in the container. In addition, number of adults, male and female were counted when they were emerged.

## DATA ANALYSIS

Nominal logistic regression was used to determine whether gender (male, female and nymph) and nutritional status influenced harbourage colour choice. Two-choice and three-choice preference data were analysed using the chi-square analysis. The statistical analysis was performed by using SPSS version 26 (IBM Corp., Armonk, NY, USA).

One-way analysis of variance was used to determine the proportion of eggs that was deposited on each coloured harbourage within each replicate. Means of eggs on each coloured harbourage were separated using student’s *t*-test.

Two-way analysis of variance (ANOVA) was performed to determine the significant difference between live individuals for each week for the rearing population. One-way ANOVA was performed to determine the significance difference between the population after 12 weeks.

## RESULTS

### Colour Choice Assays

**Figure 3:**
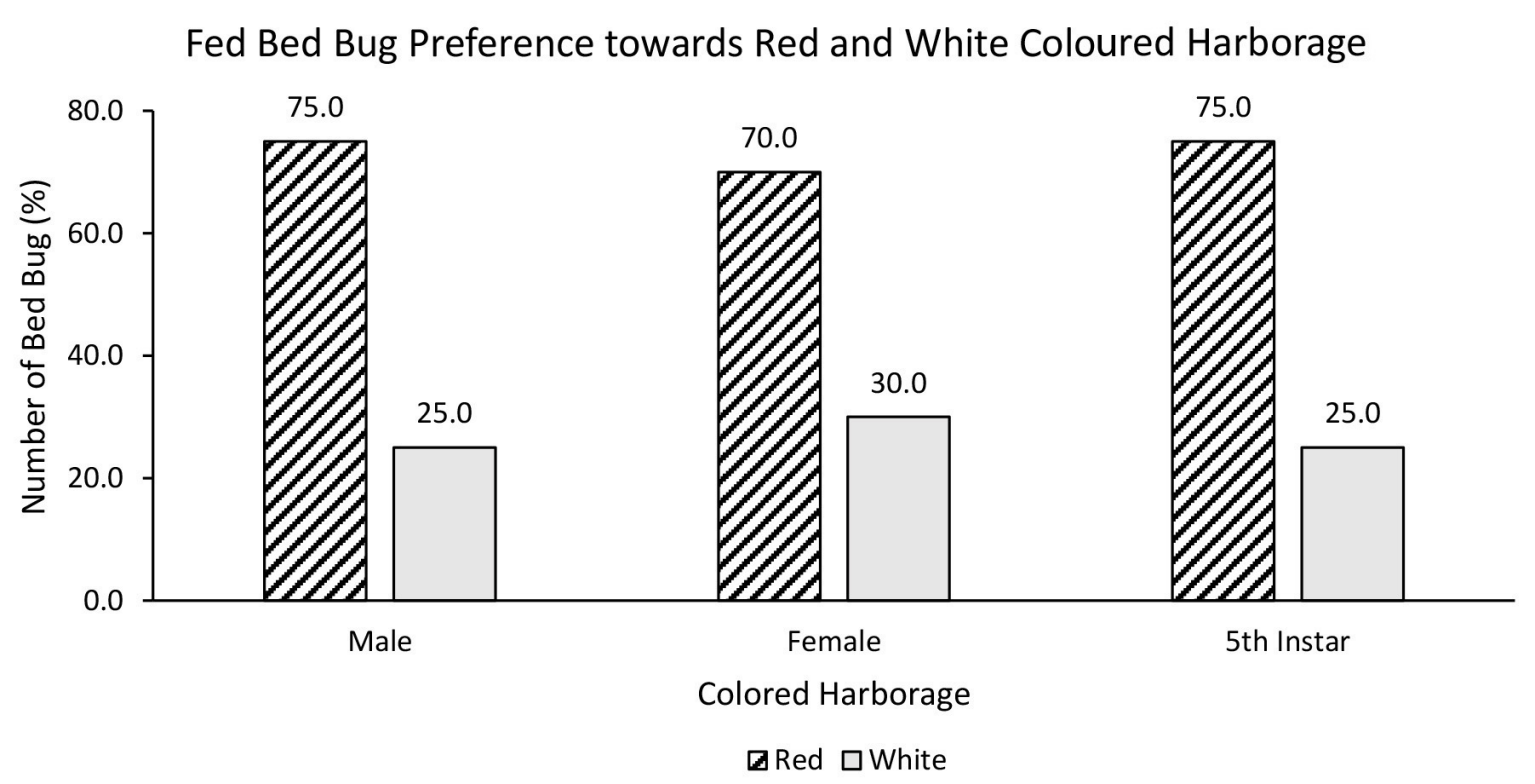
The responses of fed bed bugs to red or white coloured harbourages.

**Figure 4:**
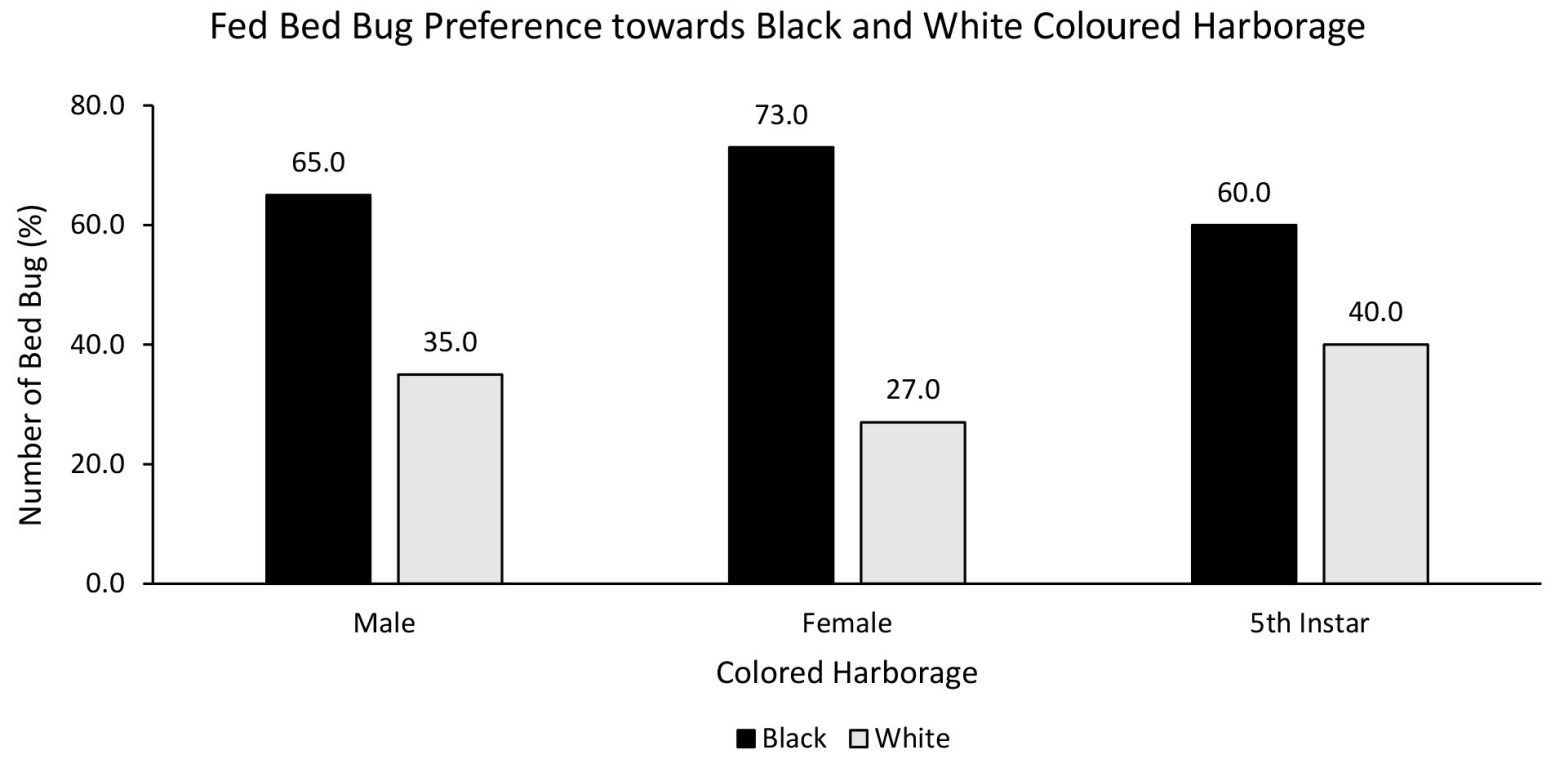
The responses of fed bed bugs to black or white coloured harbourages.

**Figure 5:**
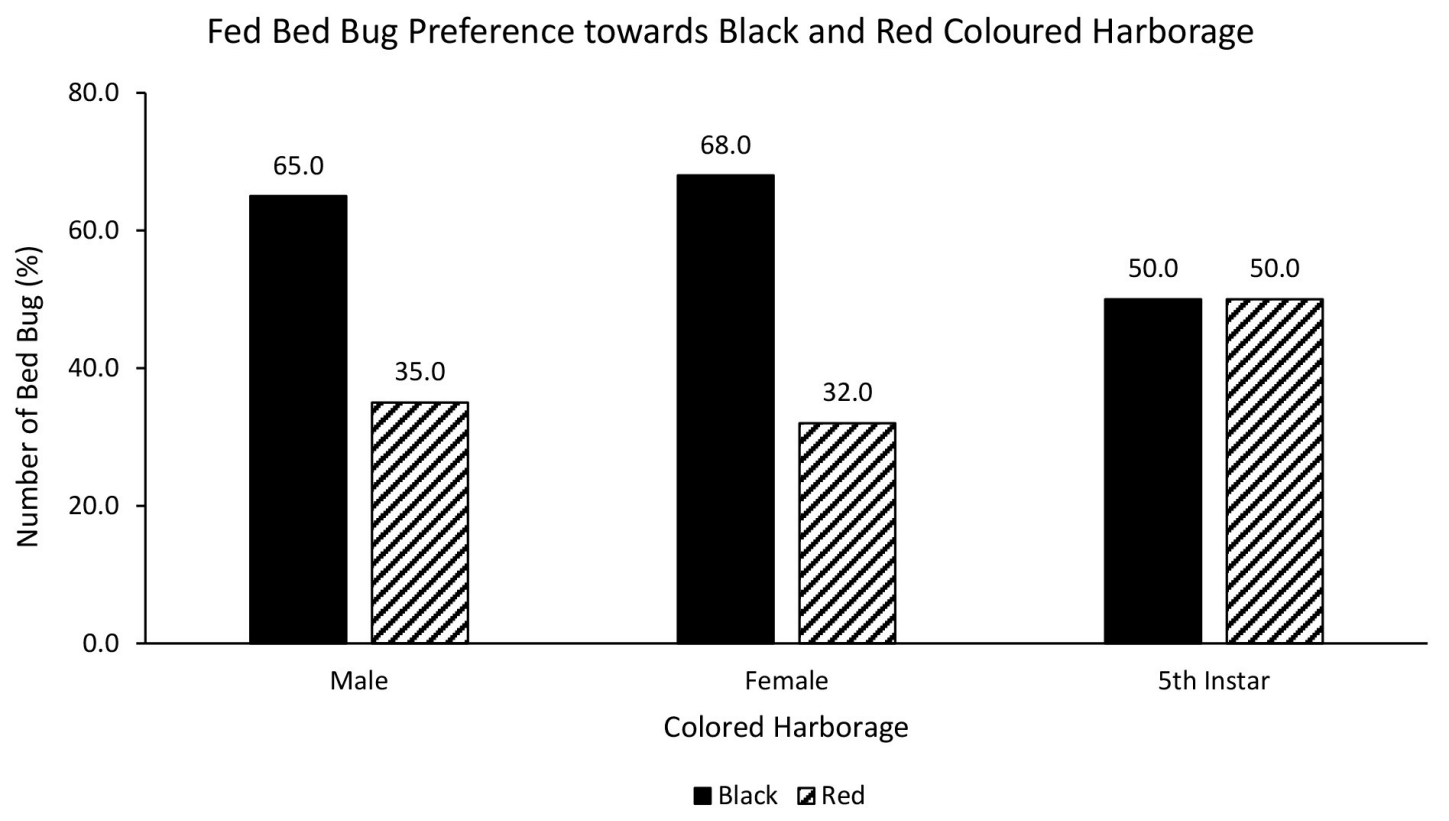
The responses of fed bed bugs to black or red coloured harbourages.

A chi-square test was performed to evaluate the relationship between bed bug’s nutritional status and preferred harbourage colour. There was no statistically significant relationship between fed bed bugs and choice of red or white coloured harbourage [χ^2^ = 0.752, df = 2, *p* = 0.686]. Fed male (75%), female (70%) and nymph (75%) strongly preferred red over white coloured harbourage (Fig. 3). There was also no statistically significant relationship between fed bed bugs and choice of black or white coloured harbourage [χ^2^ = 5.236, df = 2, *p* = 0.073]. Fed male (65%), female (73%) and nymph (60%) preferred black over white coloured harbourage (Fig. 4). On the other hand, the chi-square test yielded no statistically significant relationship between fed bed bugs and choice of black or red coloured harbourage [χ^2^ = 2.500, df = 2, *p* = 0.287]. Fed male (65%) and female (68%) showed strong preference towards black coloured harbourage compared to red coloured harbourage while fed nymph showed similar (50%) preference towards both red and black harbourages (Fig. 5).

**Figure 6:**
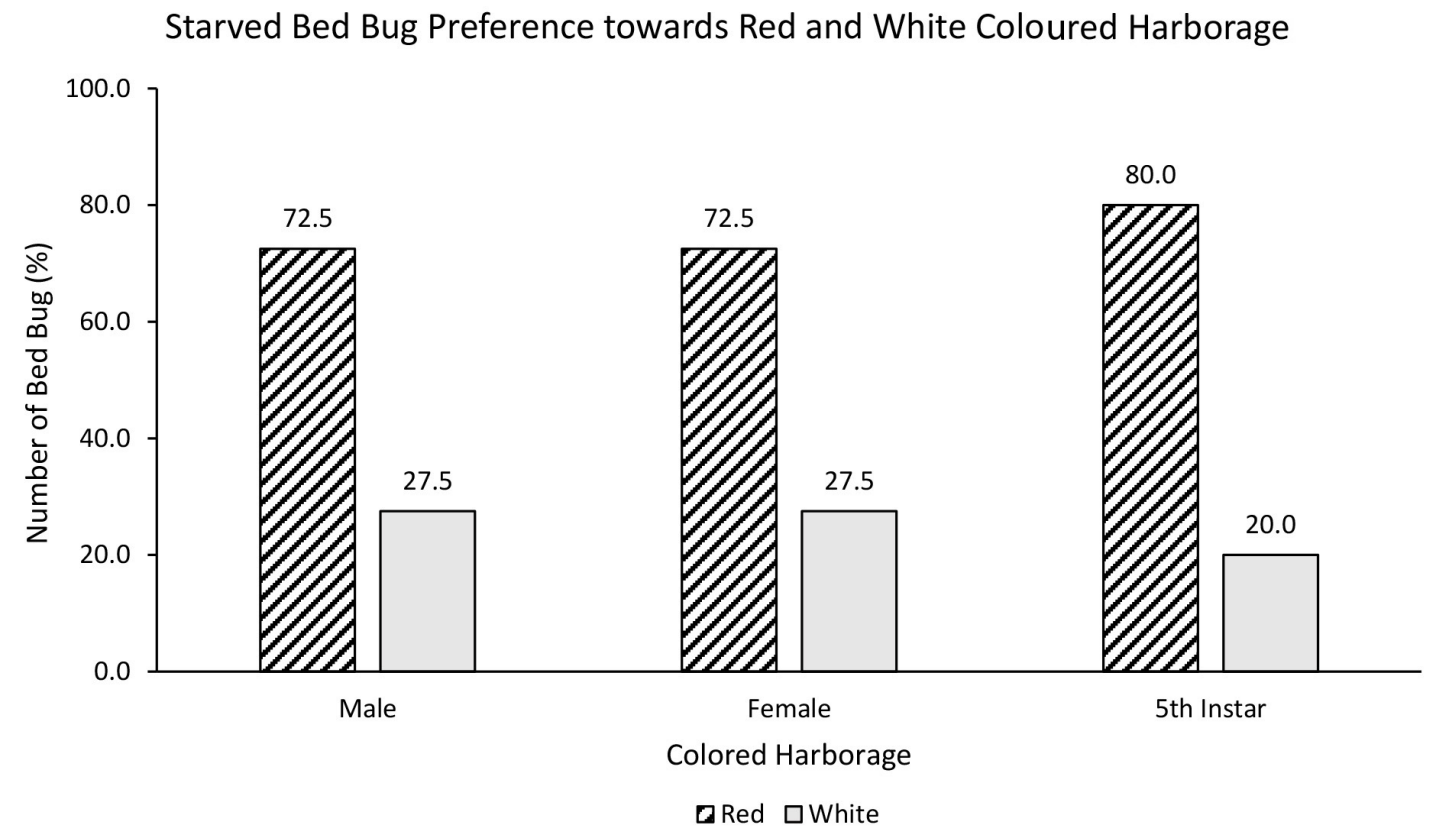
The responses of starved bed bugs to red or white coloured harbourages.

**Figure 7:**
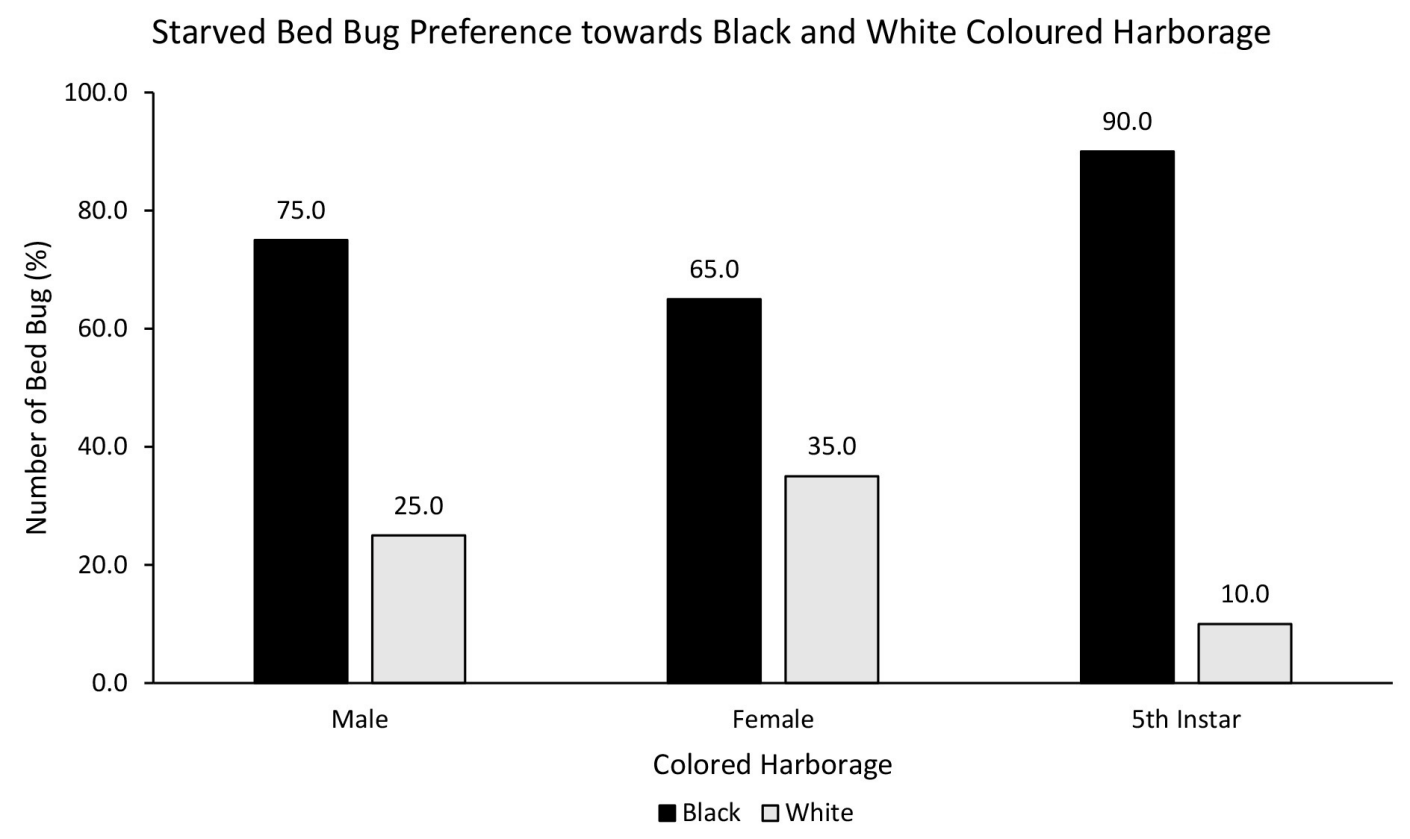
The responses of starved bed bugs to black or white coloured harbourages.

**Figure 8:**
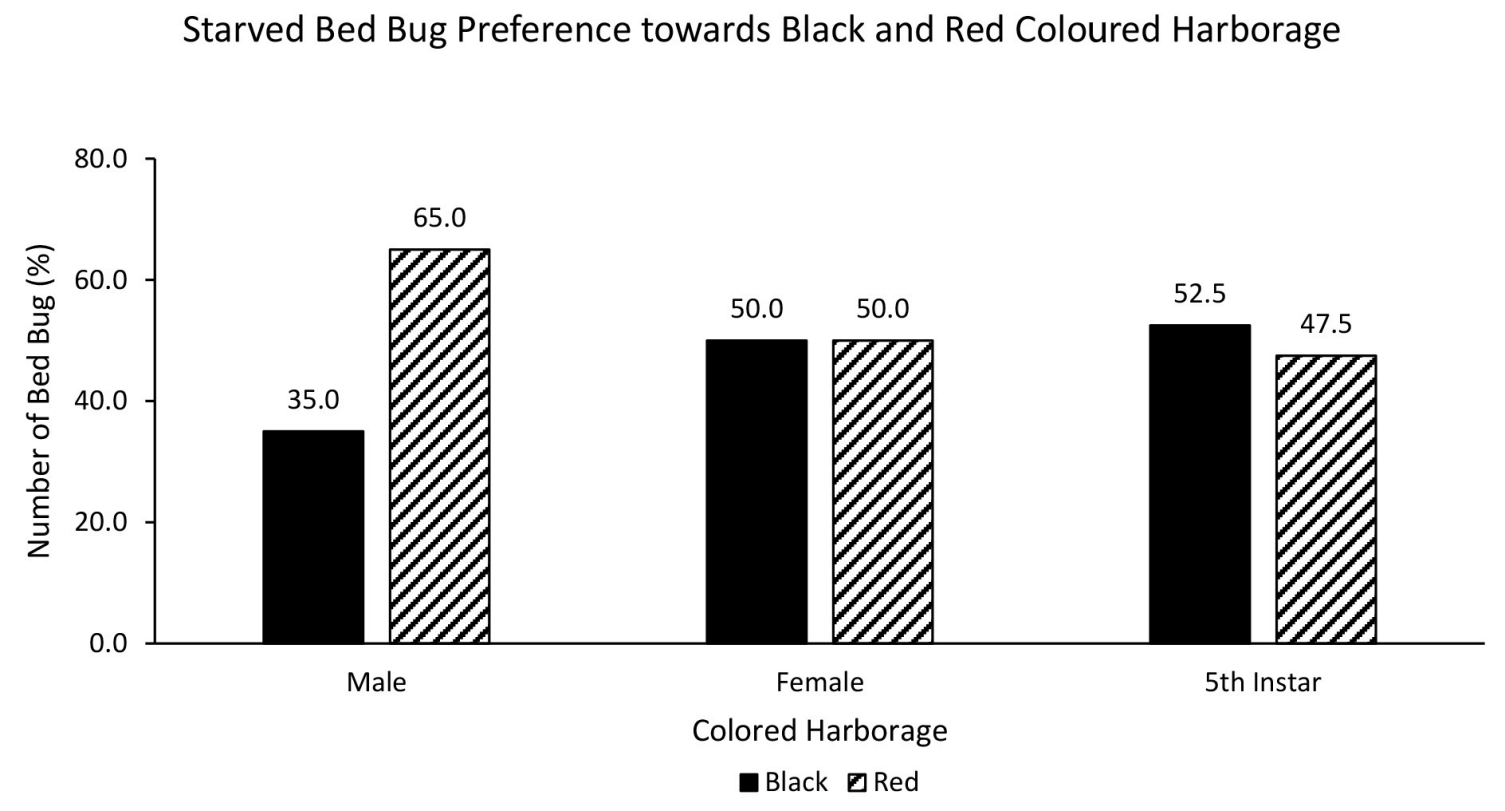
The responses of starved bed bugs to black or red coloured harbourages.

Similarly, starved male (72.5%), female (72.5%) and nymph (80%) also showed strong preference towards red over white coloured harbourage (Fig. 6), although there was no statistically significant relationship between starved bed bugs and choice of red or white coloured harbourage [χ^2^ = 0.800, df = 2, *p* = 0.670]. Interestingly, there was a statistically significant relationship between starved bed bugs and choice of black or white coloured harbourage [χ^2^ = 7.081, df = 2, *p* = 0.029]. Starved male (75%), female (65%) and nymph (90%) showed strong preference towards black over white coloured harbourage (Fig. 7). There was no statistically significant relationship between starved bed bugs and choice of black or red coloured harbourage [χ^2^ = 2.887, df = 2, *p* = 0.236]. Starved nymph showed a preference for black (52.5%) over red coloured harbourage while starved female showed similar (50%) preference towards black and red coloured harbourages, and starved male bed bug preferred red (65%) over black coloured harbourage (Fig. 8).

**Figure 9:**
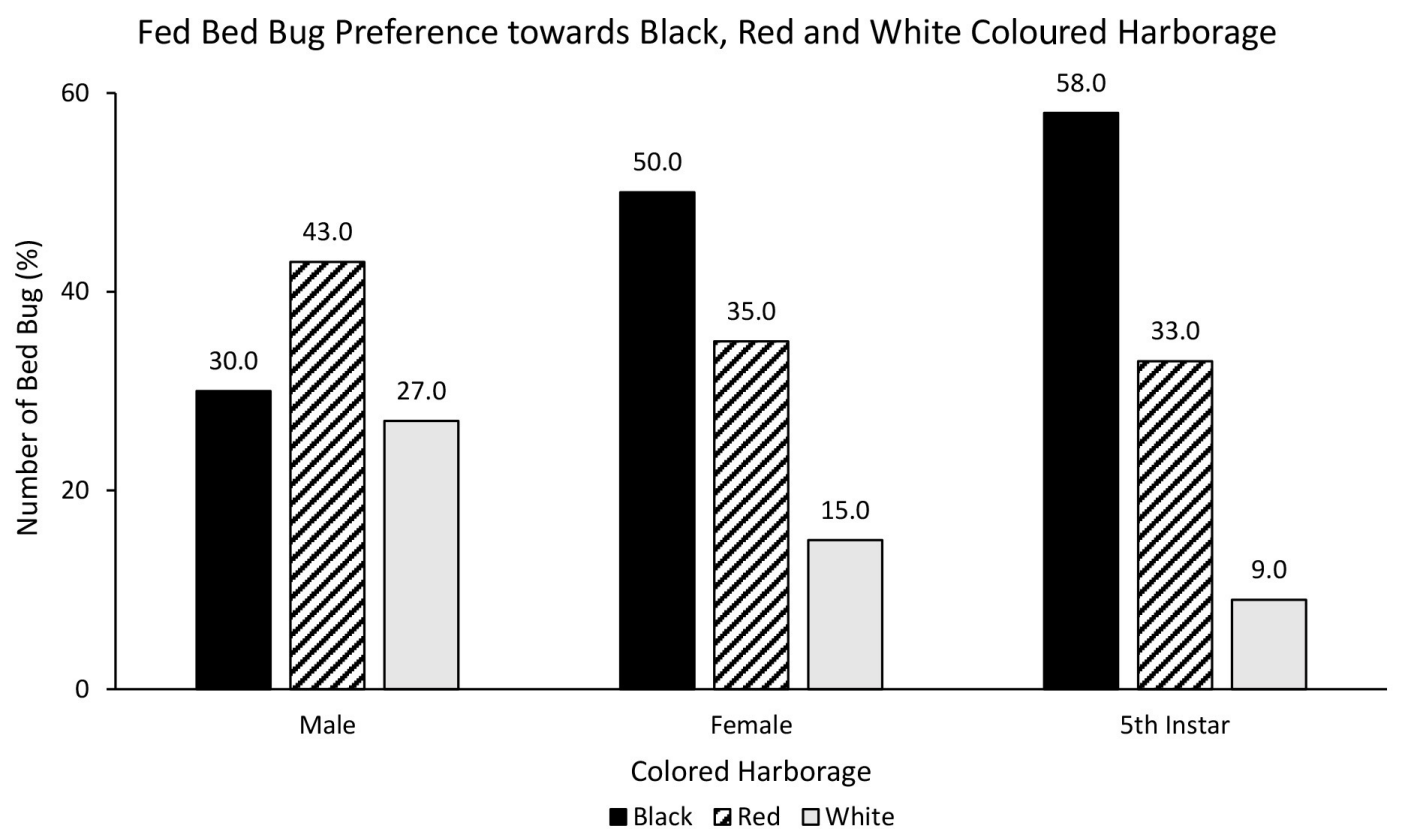
The response of fed bed bugs to black, red and white coloured harbourages.

**Figure 10:**
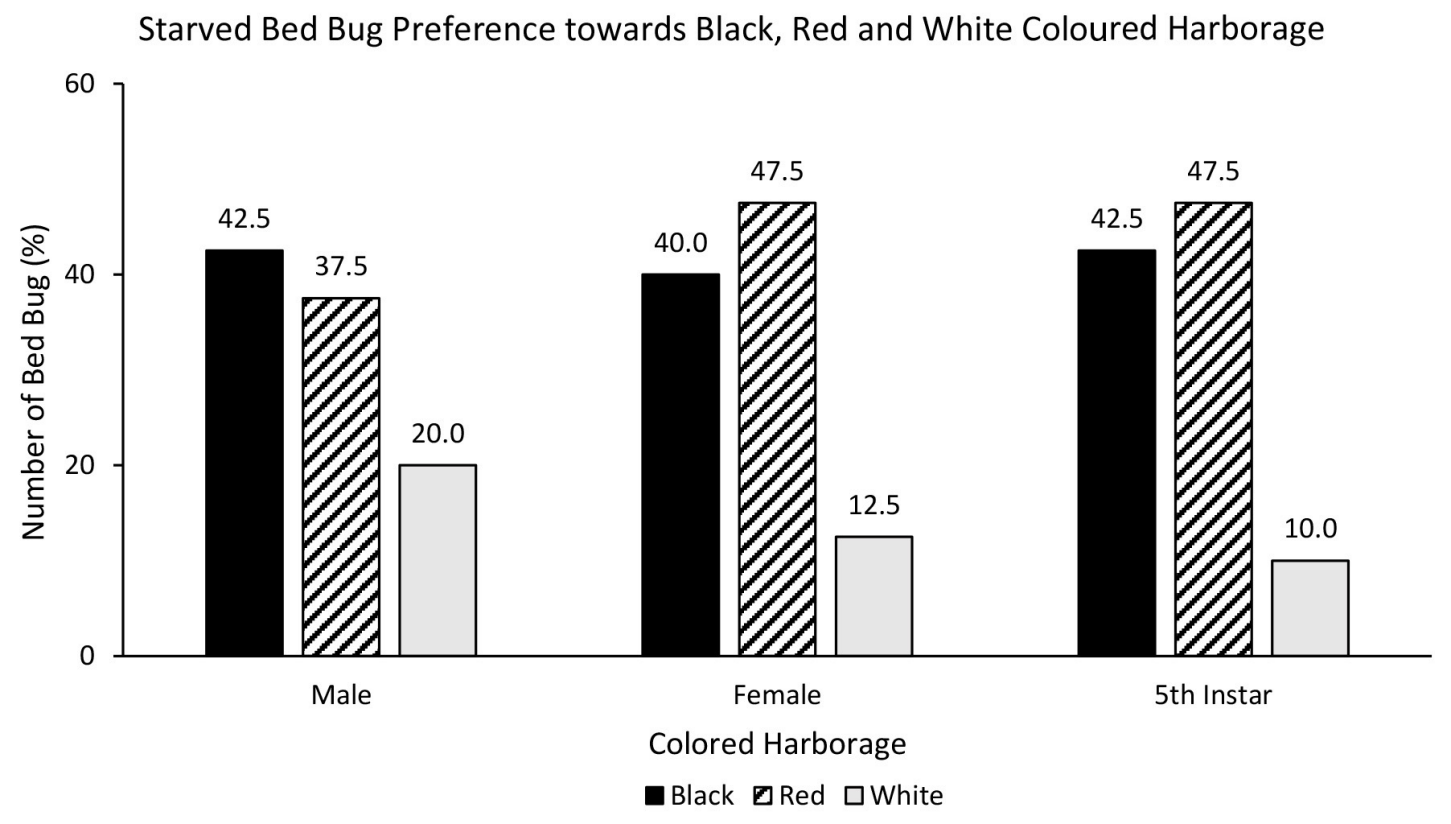
The response of starved bed bugs to black, red and white coloured harbourages.

Three choice colour assays showed that white coloured harbourage is the least preferred harbourages. However, there was no statistically significant relationship between fed bed bugs and choice of coloured harbourage [χ^2^ = 7.832, df = 4, *p* = 0.098]. Fed female (50%) and nymph (58%) strongly preferred black over red and white (Fig. 9). On the contrary, only fed male preferred red (43%) over black (30%) and white (27%) coloured harbourage. There was also no statistically significant relationship between starved bed bugs and choice of coloured harbourage [χ^2^ = 2.173, df = 4, *p* = 0.704]. Starved male preferred black (42.5%) over red (37.5%) and white (20%) coloured harbourage (Fig. 10). Starved female (47.5%) and nymph (47.5%) showed a preference towards red over black and white coloured harbourages.

### Oviposition Assays

**Figure 11:**
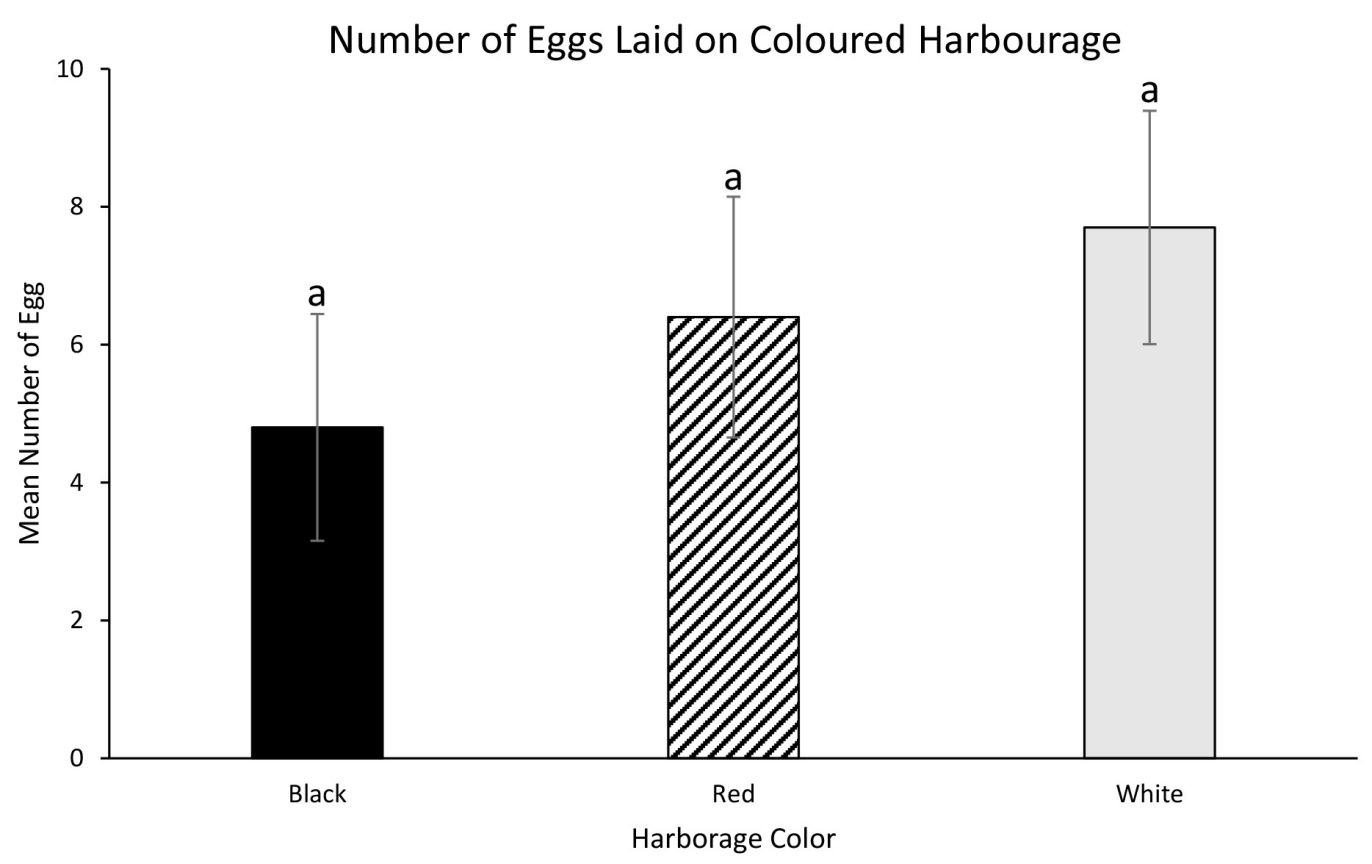
Mean of eggs laid on different coloured harbourages.

A one-way ANOVA was performed to evaluate the relationship between coloured harbourage and the proportion of eggs that was deposited on each coloured harbourage. Mean of eggs laid on black coloured harbourage was 4.8 ± 1.645, followed by eggs laid on red coloured harbourage with 6.4 ± 1.746, and white coloured harbourage recorded the highest eggs laid with 7.7 ± 1.693 (Fig. 11). The ANOVA was not significant at the 0.05 level, *F* (2, 27) = 0.734, *p* = 0.489. A post hoc Tukey HSD test also indicated there were no significant differences between the mean eggs laid on black and red coloured harbourage (*p* = 0.784), between black and white coloured harbourage (*p* = 0.458) or between red and white coloured harbourage (*p* = 0.851).

### Rearing using Coloured Harbourages

Tropical bed bugs were fed once every week on arm of a human volunteer for 12 weeks. All stages of the tropical bed bug remained attached to the parafilm membrane until the completion of their blood meals. Adult tropical bed bug emerged as early as week 6 for all types of harbourage colour. However, the number of males and females were different for all replicates with one replicate of the black harbourage recorded the highest number of females (32 individuals) and the lowest number (18 individuals) of female recorded by one replicate of black and white harbourage. After 12 weeks, all containers have various life stages, from egg to all nymphal stages and adults.

**Figure 12:**
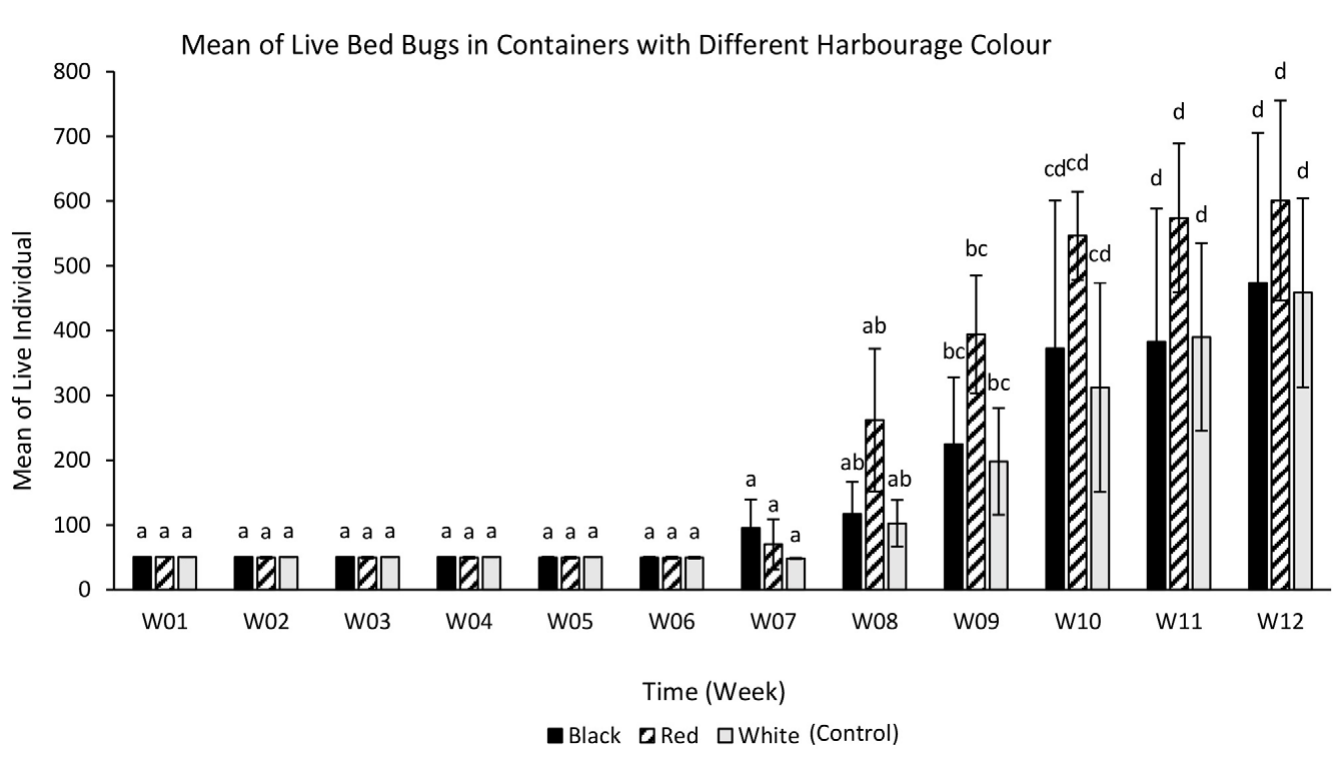
Mean of live bed bugs in the containers containing different harbourage colour.

A two-way ANOVA was performed to determine the effect of harbourage colour and time (in weeks) on the live individuals recorded in each week (Fig. 12). There was a statistically significant interaction between the effects of harbourage colour and time on the live individuals, *F* (22, 72) = 1.017, *p* = 0.457. However, there was no statistically significant difference in the mean of live individuals between week (*p* = 0.000) and between harbourage colour (*p* = 0.001). The mean of live individuals from the first week until the seventh week showed no significant difference. From the eighth to the tenth week, there was a significant difference of live individuals. Lastly, there was no significant difference of live individuals on the last two weeks.

**Figure 13:**
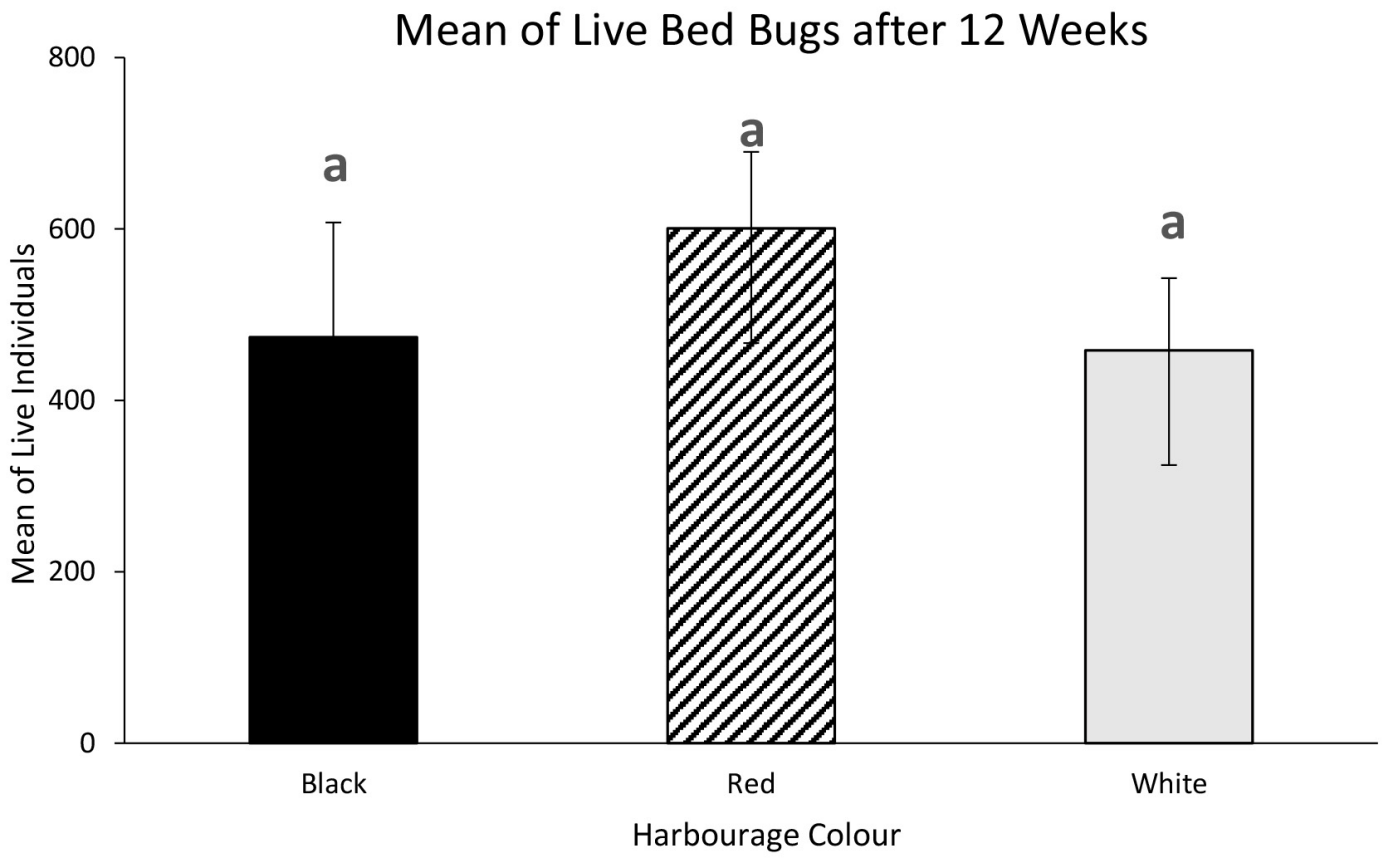
Mean of live individuals after 12 weeks according to the harbourage colour.

The initial number of individuals for all types of harbourage colour were 50 newly hatched first instars per replicate. One-way ANOVA was conducted to determine the effects of different harborage colour on the live individuals after 12 weeks of feeding (Fig. 13). There was no statistically significant difference in the number of live individuals when the tropical bed bugs were reared in containers with different harbourage colour, *F* (2, 6) = 0.555, *p* = 0.601. Containers with red coloured harbourage recorded the highest population size (600 ± 89.238), followed by black coloured habourage (473 ± 133.841) and white coloured harbourage (458 ± 84.234).

## DISCUSSION

Bed bugs can be found worldwide because they are easily transported on or in luggages, furnitures, boxes and clothes. These thin and tiny insects can be found in residential houses, hotels and public transports, and they have sparked major concerns to the hospitality and tourism industry. Bed bugs are mostly active during the night, however more significant activities has been observed under low light condition compared to under complete dark condition (Singh *et al*. 2015). Romero *et al*. (2010) explained that this peak of activity was because of the bed bug’s harbourage-seeking behaviour. Therefore, understanding the physiological colour preferences of bed bugs under lighted conditions is important, especially for infestations in human habitations where the light–dark cycles are neither necessarily dictated by natural night–day cycles, nor occur gradually.

A study conducted by McNeill *et al*. (2016) found that common bed bugs (*C. lectularius)* have different colour preferences for their harbourage and oviposition sites. Similarly, our results showed that tropical bed bugs (*C. hemipterus*) both fed and starve strongly preferred black and red coloured harbourage compared to white coloured harbourage. Fed males and females preferred black over red coloured harbourage. On the other hand, when starved, male bed bugs preferred red over black coloured harbourage. Nymph, however, did not showed preferences towards red or black harbourage, either fed or starved. Colour is also an important clue that hematophagous insects use to seek shelter (Steverding & Troscianko 2004). They documented that traps with a blue exterior and black and red interior were very effective at attracting and optimising tsetse fly trap captures. Similarly, a study by Hoel *et al*. (2007) and Mann *et al*. (2009) recorded that *Phlebotomus papatasi* and *Lutzomyia shannoni* have been shown to be attracted to red colour. Studies by Singh *et al*. (2015) have also shown that aggregations of specifically adult male bed bugs and third–fifth-instar bed bugs prefer black and red harbourages compared with other tested colours.

Both adult and immature bed bugs are able to differentiate between different colours, and preferentially select harbourages based on colour-specific visual cues. Typically, bed bugs tend to feed and find harbourages during dark periods, so colour preferences may not be important in those cases (Singh *et al*. 2015). However, these harbourage colour bioassays indicate the important role that light plays for bed bugs as they locate a suitable hiding or nesting area. Harbourages (crack and crevices) are very important to bed bugs because they spend 90% of their time in harbourages, and when not in a harbourage, they are either actively searching for a host or looking for new harbourage sites (Pinto *et al*. 2007). It has been speculated that a bed bug would go to any harbourage in an attempt to hide. However, these colour experiments show that bed bugs do not hide in just any harbourage; rather they will select a harbourage based on its colour when moving in the light.

McNeil *et al*. (2016) theorised that specific colours may represent an opportune oviposition site and mating arena or for safety purpose due to the presence of other animals and limits the visibility of predators. Oviposition assays showed that female bed bugs laid more eggs on red coloured harbourages compared to black coloured harbourages. Black (and blue) colours were also found to be the preferred oviposition sites for other insect species such as the Mediterranean fruit fly *Ceratitis capitata* and the Asian tiger mosquito *Aedes albopictus* (Katsoyannos *et al*. 1986; Hoel *et al*. 2011). Although bed bugs will oviposit their eggs on a wide range of coloured harbourage, they will avoid ovipositing under green coloured harbourages (McNeill *et al*. 2016). This avoidance behaviour may be because of green or yellow colours are usually associated with an outdoor environment having plants and bright light, which is not where bed bugs are normally found.

Bed bugs that have been reared in the containers with red paper as their harbourage recorded the highest number of individuals compared to black and white paper after 12 weeks. The life span of bed bugs are significantly influenced by their diet and mating frequencies. We fed our bed bug colonies once every week (7 days interval) because this is the optimal interval for bed bug feeding session. Bed bugs will hide in their harbourage after feeding on their host for molting and reproduction. Usually it takes about 6 to 7 days for molting process to complete and female adults typically able to lay eggs up to 10 days. Although bed bug can live up to months without blood meal, they would not be able to molt or produce offspring when they did not have a blood meal as blood is required for those process to happened. These conditions are not favourable to the researchers who need lots of bed bugs colonies for their tests and experiments. Our preliminary study which to compare two feeding interval (7 days vs. 14 days) on the reproduction of bed bugs showed that 14 days feeding interval would took longer time to produce large scale bed bug colonies in the laboratory.

In addition, we started our colonies with 50 first instars for each replicate. After 6 weeks, the fifth instars started to molt and emerged as adults. However, the number of males and females were different for each replicate. This seem to have an effect towards the reproduction rate of the tropical bed bugs. For example, replicate with higher number of females (i.e., 32 females and 18 males) would produced more offsprings (> 500) compared to replicate with lower number of females (i.e., 18 females and 28 males) which produced less offsprings (< 200) after 12 weeks of observation. Bed bug reproduction process involved very unique process called traumatic insemination where the male pierces the female’s abdomen (Harlan 2006; Reinhardt & Siva-Jothy 2007; Usinger-1966). This process caused damages to the female body. This could explain why colonies with more males than females produced less offspring. Female bed bugs would have more traumatic inseminations from more than one male which would later damages the female body and affect their ability to produce and lay eggs. On the other hand, colonies where a lot of females rather than male, produced more offsprings as they only received one traumatic insemination after each feeding session.

## CONCLUSION

Tropical bed bugs have different colour preferences for their harbourage and oviposition sites which influence by gender and nutritional status. Black and red coloured harbourages seem to be the most attractive harbourages. These findings indicate that bed bugs may have a mechanism to discriminate colours and should be useful in bed bug trap design as an attempt to enhance trap captures. Futher studies may include other colours and prolong the rearing experiment.

## References

[b1-tlsr-35-2-271] Ab Majid AH, Zahran Z (2015). Resurgence of tropical bed bug, *Cimex hemipterus* (Hemiptera: Cimicidae) infestation in Malaysia: Control strategies and challenges faced by urban pest control operator (PCO). Journal of Entomology and Zoology Studies.

[b2-tlsr-35-2-271] Ab Majid AH, Zahran Z (2017). Tropical bed bug infestation dynamics in Malaysia: Evaluation of insecticide resistance level and susceptibility on pyrethroid and carbamate insecticides (Hemiptera: Cimicidae).

[b3-tlsr-35-2-271] Abd Rahim AH, Ab Majid AH, Ahmad AH (2015). Laboratory rearing of *Cimex hemipterus* F. (Hemiptera: Cimicidae) feeding on different types of human blood compositions by using modified artificial feeding system. Asian Pacific Journal of Tropical Disease.

[b4-tlsr-35-2-271] Abd Rahim AH, Ahmad AH, Zahran Z, Ab Majid AH (2016). Human skin reactions to bites of tropical bed bug, *Cimex hemipterus* F. (Hemiptera: Cimicidae): A case study. Asian Pacific Journal of Tropical Disease.

[b5-tlsr-35-2-271] Abd Rahim AH, Zahran Z, Ab Majid AH (2014). Survey of bed bugs, Cimex hemipterus (Fabricius) infestation in Malaysia.

[b6-tlsr-35-2-271] Aboul-Nasr AE, Erakey MAS (1969). The effect of light reactions upon the bed bug *Cimex lectularius* L. Bulletin de la Société entomologique d’Égypte.

[b7-tlsr-35-2-271] Cuthill IC, McGraw KJ, Hill GE (2006). Color perception. Bird coloration: Mechanisms and measurements.

[b8-tlsr-35-2-271] Harlan HJ (2006). Bed bugs 101: The basics of *Cimex lectularius*. American Entomologist.

[b9-tlsr-35-2-271] Hoel DF, Butler JF, Fawaz EY, Watany N, El-Hossary SS, Villinski J (2007). Response of phlebotomine sand flies to light-emitting diode-modified light traps in southern Egypt. Journal of Vector Ecology.

[b10-tlsr-35-2-271] Hoel DF, Obenauer PJ, Clark M, Smith R, Hughes TH, Larson RT, Diclaro JW, Allan SA (2011). Efficacy of ovitrap colors and patterns for attracting *Aedes albopictus* at suburban field sites in north-central Florida. Journal of the American Mosquito Control Association.

[b11-tlsr-35-2-271] How YF, Lee CY (2014). Effects of temperature and humidity on the survival and water loss of *Cimex hemipterus* (Hemiptera: Cimicidae). Journal of Medical Entomology.

[b12-tlsr-35-2-271] Katsoyannos BI, Panagiotidou K, Kechagia I (1986). Effect of color properties on the selection of oviposition site by *Ceratitis capitata*. Entomologia Experimentalis et Applicata.

[b13-tlsr-35-2-271] Lim L, Ab Majid AH (2020). Metagenomic 16S rDNA amplicon data of microbial diversity of guts of fully fed tropical bed bugs, *Cimex hemipterus*(F.) (Hemiptera: Cimicidae). Data in Brief.

[b14-tlsr-35-2-271] Mann RS, Kaufman PE, Butler JF (2009). Lutzomyia spp. (Diptera: Psychodidae) response to olfactory attractant and light emitting diode modified Mosquito Magnet X (MM-X) traps. Journal of Medical Entomology.

[b15-tlsr-35-2-271] McNeill CA, Pereira RM, Koehler PG, McNeill SA, Baldwin RW (2016). Behavioral responses of nymph and adult *Cimex lectularius* (Hemiptera: Cimicidae) to colored harborages. Journal of Medical Entomology.

[b16-tlsr-35-2-271] Menzel R, Backhaus W, Gouras P (1991). Color vision in insects. Vision and visual dysfunction: The perception of color.

[b17-tlsr-35-2-271] Miller DM, Polanco AM, Rogers J (2013). Bed bug biology and behavior.

[b18-tlsr-35-2-271] Pinto LJ, Cooper R, Kraft SK (2007). Bed bug handbook: The complete guide to bed bugs and their control.

[b19-tlsr-35-2-271] Reinhardt K, Siva-Jothy MT (2007). Biology of the bed bugs (Cimicidae). Annual Review of Entomology.

[b20-tlsr-35-2-271] Romero A, Potter MF, Haynes KF (2010). Circardian rhythm of spontaneous locomotor activity in the bed bug *Cimex lectularius* L. Journal of Insect Physiology.

[b21-tlsr-35-2-271] Singh N, Wang C, Cooper R (2015). Role of vision and mechanoreception in bed bug, *Cimex lectularius* L. behavior. PLoS One.

[b22-tlsr-35-2-271] Steverding D, Troscianko T (2004). On the role of blue shadows in the visual behavior of tsetse flies. Proceedings of the Royal Society B: Biological Sciences.

[b23-tlsr-35-2-271] Usinger RL (1966). Monograph of cimicidae.

[b24-tlsr-35-2-271] Weiss HB (1943). Color perception by insects. Journal of Economic Entomology.

[b25-tlsr-35-2-271] Zahran Z, Ab Majid AH, Abd Rahim AH, Ahmad AH (2016). A survey on the infestation levels of tropical bed bugs in Peninsular Malaysia: Current updates and status on resurgence of *Cimex hemipterus* (Hemiptera: Cimicidae). Asian Pacific Journal of Tropical Disease.

[b26-tlsr-35-2-271] Zorrilla-Vaca A, Silva-Medina MM, Escandón-Vargas K (2015). Bedbugs, Cimex spp.: Their current world resurgence and healthcare impact. Asian Pacific Journal of Tropical Disease.

